# Split Tensile Strength Prediction of Recycled Aggregate-Based Sustainable Concrete Using Artificial Intelligence Methods

**DOI:** 10.3390/ma15124296

**Published:** 2022-06-17

**Authors:** Muhammad Nasir Amin, Ayaz Ahmad, Kaffayatullah Khan, Waqas Ahmad, Sohaib Nazar, Muhammad Iftikhar Faraz, Anas Abdulalim Alabdullah

**Affiliations:** 1 Department of Civil and Environmental Engineering, College of Engineering, King Faisal University, P.O. Box 380, Al-Hofuf 31982, Al-Ahsa, Saudi Arabia; kkhan@kfu.edu.sa (K.K.); 218038024@student.kfu.edu.sa (A.A.A.); 2 MaREI Centre, Ryan Institute and School of Engineering, College of Science and Engineering, National University of Ireland Galway, H91 TK33 Galway, Ireland; a.ahmad8@nuigalway.ie; 3 Department of Civil Engineering, COMSATS University Islamabad, Abbottabad 22060, Pakistan; waqasahmad@cuiatd.edu.pk (W.A.); sohaibnazar@cuiatd.edu.pk (S.N.); 4 Department of Mechanical Engineering, College of Engineering, King Faisal University, P.O. Box 380, Al-Hofuf 31982, Al-Ahsa, Saudi Arabia; mfaraz@kfu.edu.sa

**Keywords:** sustainable concrete, recycled aggregate, machine learning, decision tree, artificial neural network, random forest

## Abstract

Sustainable concrete is gaining in popularity as a result of research into waste materials, such as recycled aggregate (RA). This strategy not only protects the environment, but also meets the demand for concrete materials. Using advanced artificial intelligence (AI) approaches, this study anticipates the split tensile strength (STS) of concrete samples incorporating RA. Three machine-learning techniques, artificial neural network (ANN), decision tree (DT), and random forest (RF), were examined for the specified database. The results suggest that the RF model shows high precision compared with the DT and ANN models at predicting the STS of RA-based concrete. The high value of the coefficient of determination and the low error values of the mean absolute error (MAE), mean square error (MSE), and root mean square error (RMSE) provided significant evidence for the accuracy and precision of the RF model. Furthermore, statistical tests and the k-fold cross-validation technique were used to validate the models. The importance of the input parameters and their contribution levels was also investigated using sensitivity analysis and SHAP analysis.

## 1. Introduction

**Figure 1:**
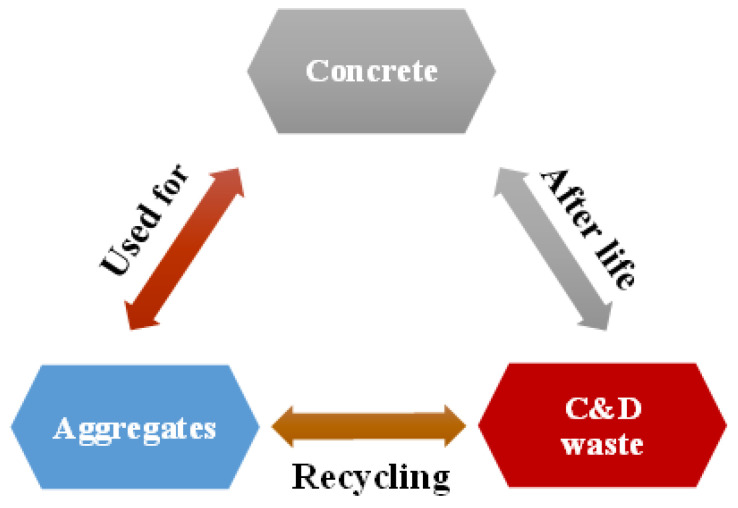
Schematic representation of the recycling process for the aggregate.

Due to the building industry’s increasing demand, the production and application of ecologically friendly concrete manufactured from waste materials have increased quickly over the past few decades [1,2,3]. Concrete production currently averages around 1 t per person per year [4]. However, the considerable amount of concrete produced meets building industry requirements and has a detrimental influence on environmental circumstances [5,6,7,8]. The manufacturing of concrete and aggregates results in the generation of carbon dioxide (CO_2_), dust, and other hazardous gases, which cause environmental damage [9,10,11,12,13,14]. Recently, the application of RA in concrete has acquired prominence in research, since it produces environmentally friendly concrete that also performs well in terms of mechanical properties [15,16,17,18]. Globally, the need for this type of concrete is increasing as a result of uncontrollable circumstances, such as earthquakes, resulting in major environmental challenges [19,20,21,22,23,24]. RA-based concrete is one of the potential options for lowering the rate of natural resource consumption [19,25,26,27]. RA takes various forms, such as tiles, different types of concrete marbles, asphalt, and brick aggregates. RA concrete refers to aggregates that are commonly refined by the parent or recycled crushing of concrete, such as waste-crushed concrete [28]. An illustrative diagram of the recycling operation is depicted in Figure 1. The concept of recycling RA from concrete containing waste materials has been used in Europe since World War II. Currently, RA concrete is employed as a sub-base material for unbound pavements. It is also employed in structural concrete [29,30]. Moreover, the concrete’s fine fraction from construction and demolishing (C&D) waste can be utilized as a partial replacement of cement [31]. This results in the reduction in cement demand, and the problems raised from the production and use of cement are controlled. The positive effects of recycling C&D waste for use in construction materials are self-evident, as it results in environmentally beneficial construction. While the application of this type of waste in concrete is limited because of its limited strength, low Young’s modulus, and higher deformation, the desired strength can be attained by applying an appropriate mix design [32].

The application of the RA in concrete can considerably enhance the characteristics of the material if additional suitable components are used. Recent years have seen a surge in the popularity of new approaches to AI for predicting the required outcomes in the area of material testing [33]. For the investigation of the strength (compressive or tensile) of concrete, since it typically takes a number of days to reach the necessary strength, without wasting time or money, AI approaches may be employed to anticipate the strength properties of concrete. Typically, AI algorithms, such as neuro-fuzzy, decision trees (DT), neural networks, bagging regressors, boosting regressors, AdaBoost, support vector machines (SVM), and gradient boosting are employed to anticipate the strength of concrete. De-Cheng et al. [34] anticipated the CS of concrete using an adaptive boosting technique using 1030 data points, achieving a 98 percent accuracy rate compared to the real results. Dong et al. [35] forecasted the performance of concrete using an ANN model and Monte Carlo simulation. Muhammad et al. [36]’s research was based on the anticipation of concrete strength with bagasse ash; the projected accuracy was over 80%, showing superior performance. Aliakbar et al. [37] used GEP to develop a novel formulation for the properties of RA concrete. They used data from the literature to find the CS, flexural strength, STS, and elastic modulus. Taihao et al. [38]’s study described the application of various ML techniques for the estimation of the Young’s modulus of concrete containing RA. Random forest (RF) and SVM were used to predict the outcome, which demonstrates the accuracy of the prediction.

In this research, the DT, ANN, and random forest (RF) techniques from AI were used to predict concrete STS containing RA. The employed models in the study were applied and compared to determine whether the model performed better in terms of result prediction. The coefficient of determination (R^2^) value was used to determine the precision level between the real and anticipated outcome, with a larger value indicating the model’s superior performance. Statistical checks for examining various errors (RMSE, MAE, and MSE) in the data were also employed to verify each model’s actual performance in predicting the STS of the RA-based concrete. In addition, K-fold cross-validation (CV) was used in the study to test the model’s performance. Furthermore, sensitivity and SHAP analyses were performed to determine the percentage contribution of all the variables and the interaction of the raw materials utilized to predict the STS for the RA-based concrete. The aim of the study was to investigate the individual and ensemble type of ML algorithms using Anaconda Navigator software for predicting the strength of selected concrete. The employed models were run on the basis of Python coding to forecast the splitting tensile strength of recycled aggregate-based (RAC) concrete. This study is also novel in that it investigated this important property using both individual (DT, ANN) and ensemble (RF) ML techniques. In the ensemble ML approach (RF), the optimization was performed by according the 20-sub models the strongest and most precise outcome. Moreover, a comparative study was also conducted between the employed algorithms for further use in predicting the STS of concrete. This study would also be useful for the area of material testing, which would not only minimize the physical effort in the laboratory but also reduce the cost and time of the project.

## 2. Methods and Database Description

**Figure 2:**
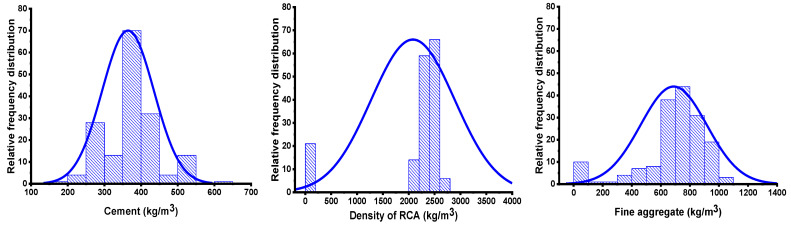

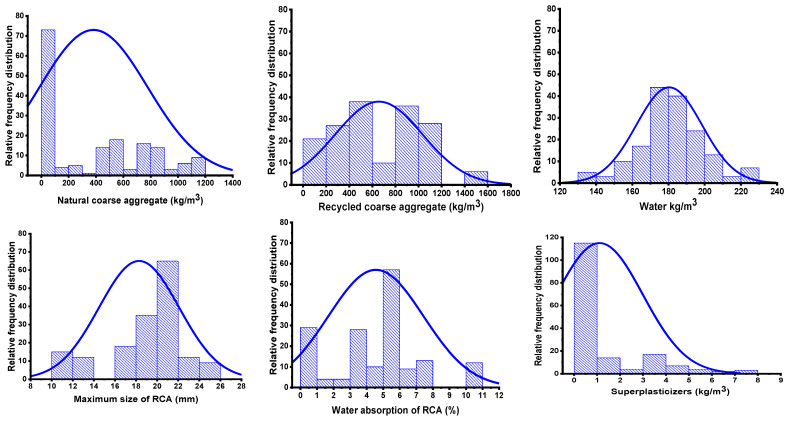
Reflection of the distribution for the input variable’s relative frequency.

**Figure 3:**
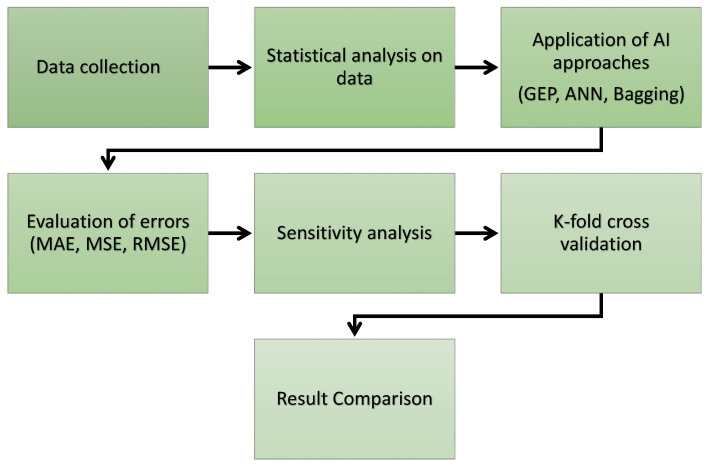
Flow chart of the research program.

The models’ performance was determined by their variables and the database required to run them. The data utilized to run the models for predicting the STS of concrete in this study were obtained from prior research [39,40,41,42,43,44,45,46,47,48,49,50,51,52,53,54,55,56,57]. The data were arranged in such a way that the model could realize both input and output parameters. A number of parameters were kept in the category of input, such as water (WA), cement (CE), fine aggregate (FA), natural coarse aggregate (NACA), recycled coarse aggregate (RECA), superplasticizers (SPS), the maximum size of RA (SRA), the density of RA (DRA), and water absorption of RA (WARA), with one output parameter (STS) used to run the models. However, the Python coding was configured in such a way that it automatically split the data into 60% for training, 20% for testing, and 20% for validation of the model. Figure 2 is the reflection of the variables’ frequency dispersal. Table 1 displays the expressive statistical analysis of the parameters, as well as the numerous mathematical descriptions that are included. Additionally, the complete adopted methodology of this study is offered in the graphical representation, as can be seen in Figure 3, which offers information on the study’s sequential methodology.

### 2.1. Random Forest (RF)

**Figure 4:**
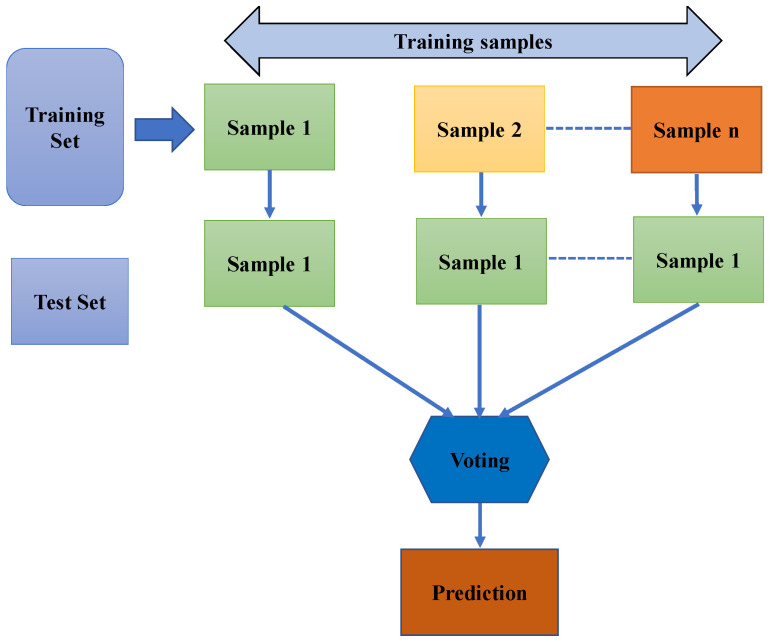
Execution process of the RF model for the required outcome.

The RF approach is immensely useful as a general-purpose classification and retrogration tool. The strategy is used to handle two distinct groups of problems: developing a prediction rule in a supervised learning problem and evaluating and ranking variables based on their predictive power. Moreover, it is flexible for larger-scale issues and adaptable to a range of ad hoc learning tasks, and it returns calculations of different levels of significance. The strategy, which aggregates the predictions of numerous randomized decision trees, outperforms other techniques when the number of variables exceeds the number of observations. There is a wide variety of RF strategies, which are defined by (1) the method employed to build each individual tree, (2) the process adopted to generate the altered data sets employed to assemble each individual tree, and (3) the process incorporated to aggregate the projections of each individual tree to create a unique consensus forecast. Additionally, it is adaptive to challenges on a big scale, capable of performing a variety of ad hoc learning tasks, and delivers measures of varying relevance. The execution method of the RF approach is shown in Figure 4.

### 2.2. Neural Network (NN)

**Figure 5:**
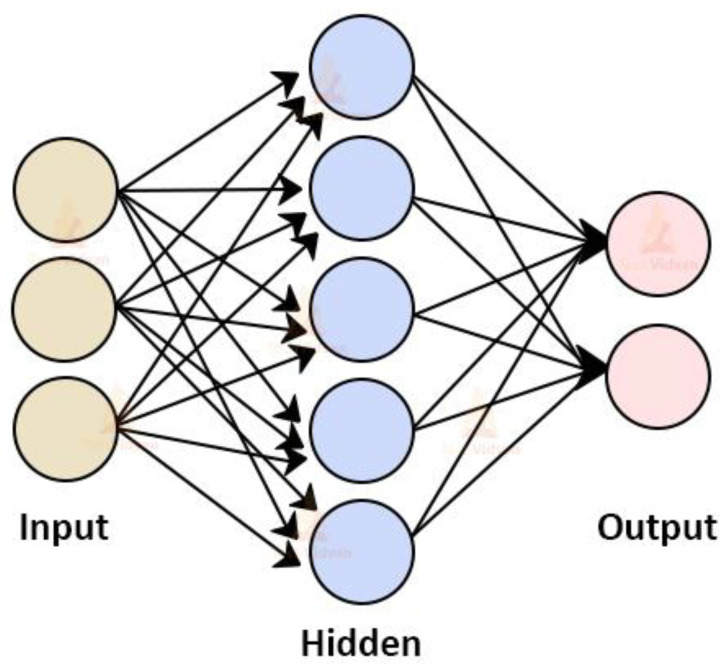
Schematic representation of the artificial neural network.

NN alludes to ensuring the type of system that is stimulated by the neural networks in biology that strengthen the brain. ANN is built on a platform of connected units or nodes, referred to as artificial neurons. Neurons’ function and structure are exact replicas of the brain. Prior to processing, these neurons receive a signal and can communicate with the neuron to which they are attached. The initial value denotes a “signal” at a joint, while each neuron’s outputs are expressed by a variety of non-linear parameters of the total sum of its inputs. As with neurons, edges often have a weight that changes as the learner develops. The weight is modified in accordance with the link’s signal strength. Suppose a neuron receives an aggregate message because of the possibility of the entry point, such as a previous frequency response. Neurons are typically structured in the form of layers. Every layer’s outputs serve a distinct purpose. These stages facilitate the transfer of signals from the first (input layer) to the last (output layer). The artificial neural network is a scalable system that employs a wholly distinct methodology from conventional AI and instruction processing technologies. It helps to reduce the limitations of previous logic-based AI in dealing with intuition and large datasets and offers the benefits of adaptive, self-organizing, and real-time learning. Figure 5 illustrates the architecture of an NN.

### 2.3. Decision Tree (DT)

**Figure 6:**
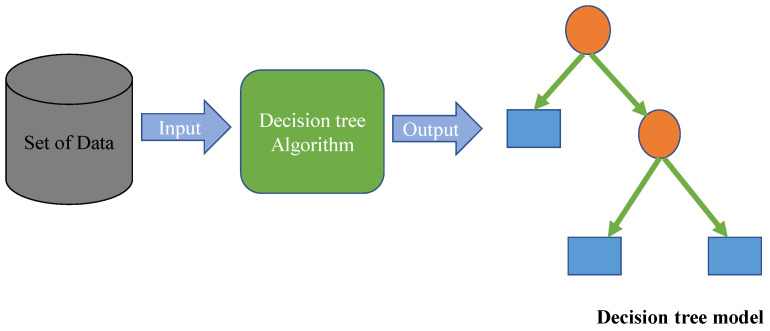
Flow chart indicating the executing process of the decision tree.

In comparison to other categorization approaches, decision trees can be generated very quickly. These trees may simply be translated to SQL queries that can be used to access databases effectively. When compared to other classification approaches, decision-tree classifiers achieve comparable, if not superior, accuracy. The decision-tree algorithm can be applied serially or in parallel, depending on the volume of data, the amount of memory available on the computer resource, and the algorithm’s adaptability. DT classifiers have not been as widely adopted as analytical or neural/connectionist approaches in the remote sensing sector. Among the obvious benefits of decision trees are their capacity to maintain data measured on a variety of scales, their lack of suppositions about the summary statistics of the data in each class, and their resilience, specifically, and capacity to deal with non-linear interactions between characteristics and classes. The primary advantage is their capacity to select the most skewed aspect and improve its comprehensibility. Additionally, they are easily classifiable and interpretable. Furthermore, they are applicable to both continuous and discrete data sets. Figure 6 depicts a schematic illustration of the DT model in more detail.

## 3. Result and Discussion

### 3.1. ANN Model Outcome

**Figure 7:**
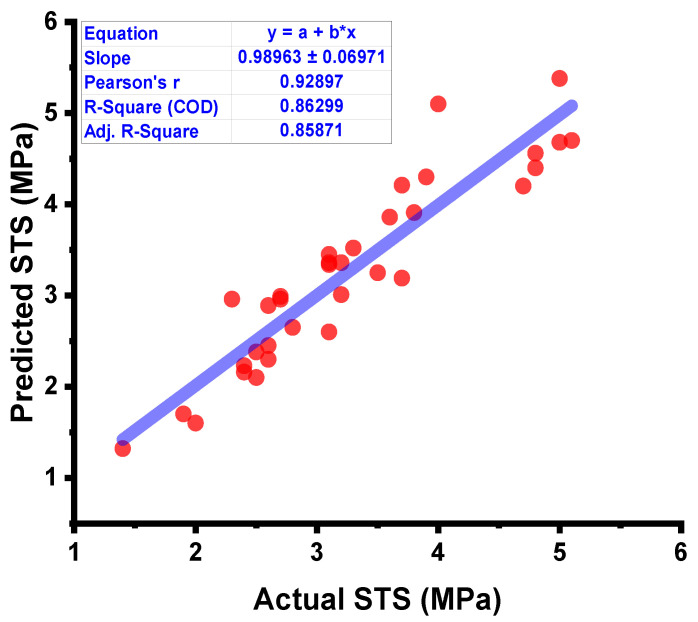
ANN model results comparison between the results from laboratory work and predictions.

**Figure 8:**
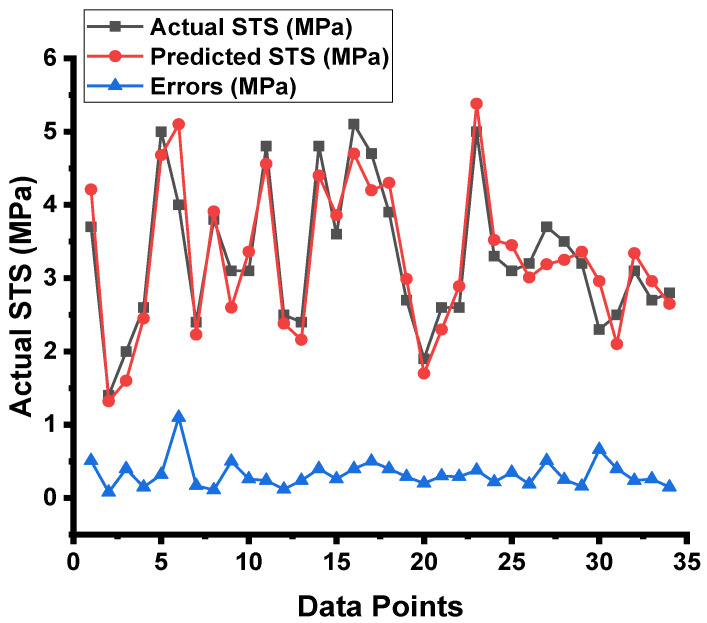
ANN model’s actual results, predictions, and dispersal of their difference.

Figure 7 illustrates the numerical calculation of the actual and potential data for the STS of the concrete material by employing the ANN model. The NN technique shows reasonably accurate outcomes, with less variation among the results collected from the laboratory work and the projected outputs from the model. With an R^2^ score of 0.86, the selected model precision is reasonably acceptable in predicting the required result. Figure 8 shows the scattering of the results obtained from the experimental approach (targeted), predictions, and error results for the ANN model. For the data set, the high, low, and average output were noted as 1.10, 0.080, and 0.320 MPa, respectively. Although 2.94% of these scores were between 0.10 and 0.30 MPa, 52.95% of the erroneous values fell between 0.10 and 0.30 MPa, and 40.11% outpaced 0.31 MPa.

### 3.2. DT Model Outcome

**Figure 9:**
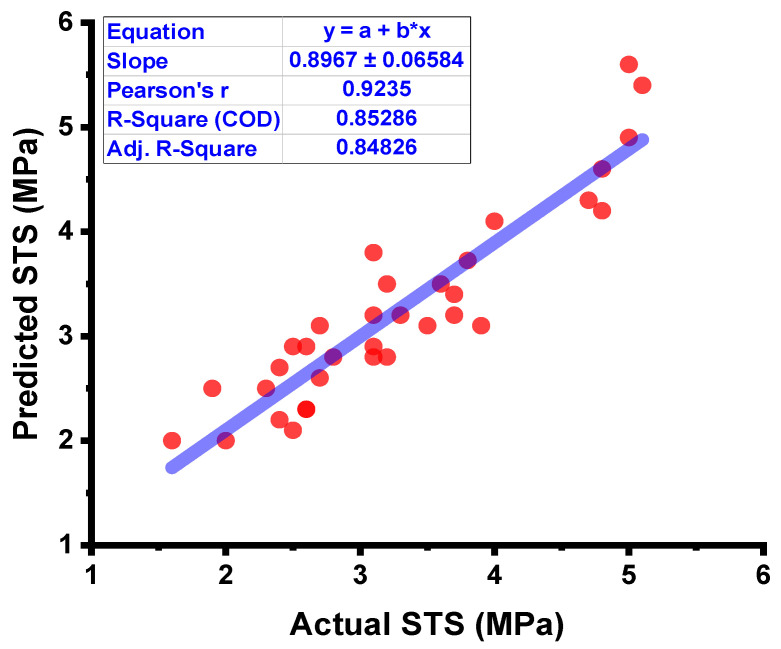
DT model results comparison between the results from laboratory work and predictions.

**Figure 10:**
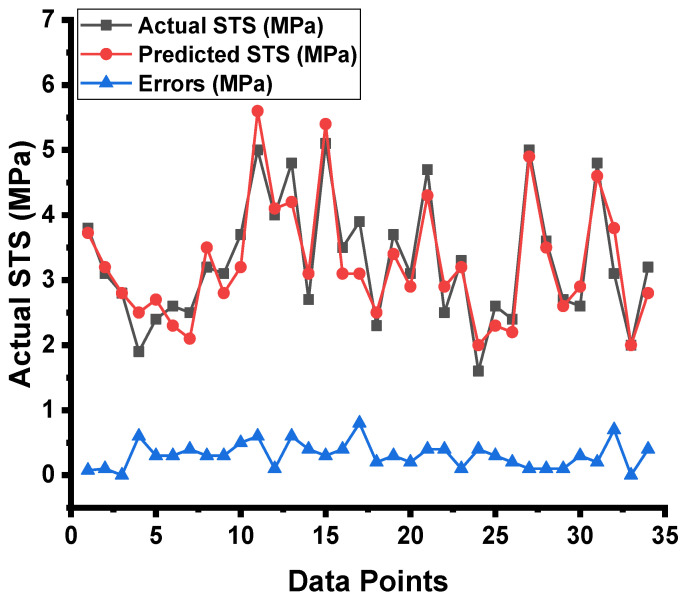
DT model’s actual results, predictions, and dispersal of their difference.

The forecasted results captured from the DT model for the strength of the concrete and the statistical assessment of the experimental and projected values are depicted in Figure 9. The DT approach produces good precision, with only a minor variation between the predicted and examined values. According to R^2^, the model is able to precisely determine the outcomes of a given experiment. The experiment aimed at expected results and the resulting data on the difference between them for the model can be seen in Figure 10. It was found that the test set’s highest and mean data were 0.8 and 0.30 MPa, respectively. While 8.82 percent of these values were up to 0.1 MPa, 28.4% were between 0.10 and 0.30 MPa, and 61.76% outpaced 0.30 MPa.

### 3.3. Random-Forest Model Outcome

**Figure 11:**
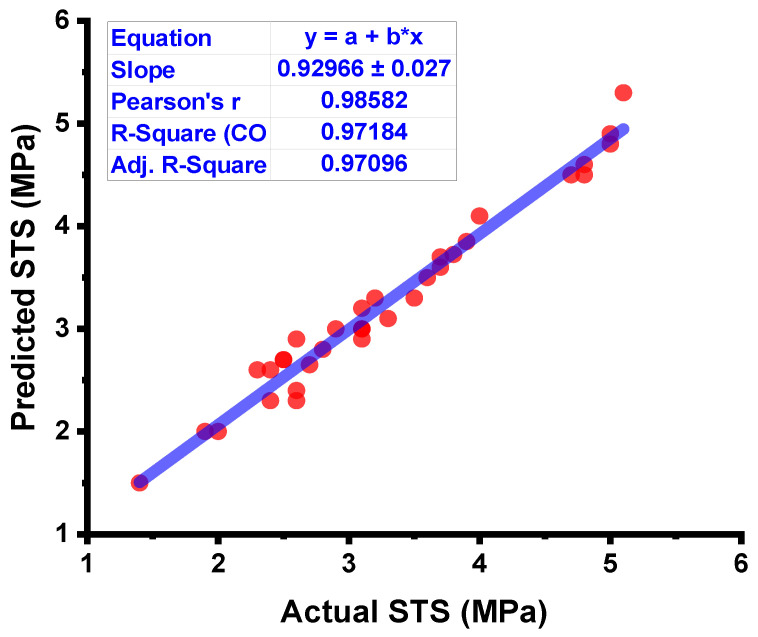
RF model result comparison between the results from laboratory work and predictions.

**Figure 12:**
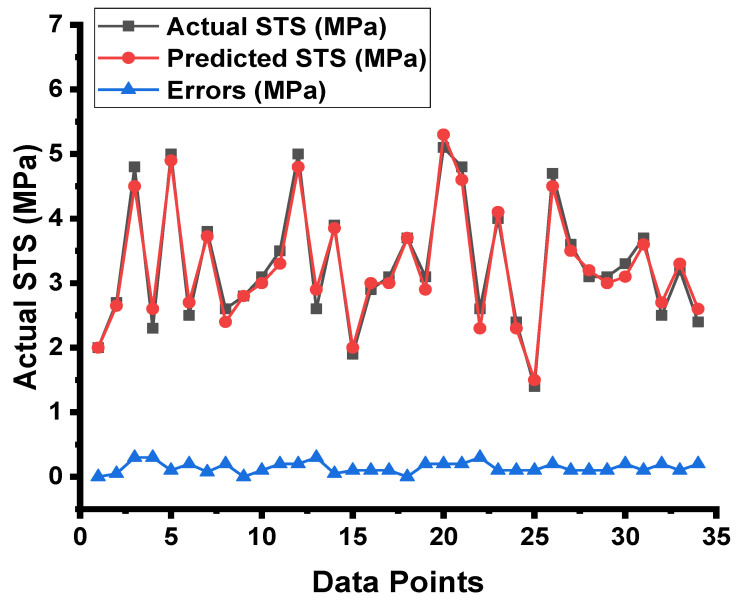
RF model’s actual results, predictions, and dispersal of their difference.

The results from the RF model are shown in Figure 11 and Figure 12. As shown in Figure 11, the RF model surpasses both the DT and the ANN model in terms of outcome accuracy, with an R^2^ value of 0.97. Figure 12 depicts the dispersal of the experimental results, the anticipated results, and the difference between them for the RF model. The maximum, minimum, and average values of the testing set were 0.30, 0, and 0.14 MPa, respectively. However, 83.33% of these results were lower than 0.1 MPa, and 70.58% were between 0.1 and 0.3 MPa. When compared to the DT and ANN models, the RF model’s strong reliability is further confirmed by the low values of the errors, which were significantly lower than those of the other models.

## 4. Cross-Validation (CV) of K-Fold

During the operation of the model, the effectiveness of the model was decided using the k-fold cross-validation (CV) approach. The k-fold CV technique is normally introduced for model validation; the procedure adopts the data selection and is spread randomly to divide the data into the ten groups. Nine sets must always be assigned for training, and one group should be assigned for validation purposes. In addition, the process must be reproduced 10 times in order to reach an average output result. CV’s lengthy process ensures the models’ high level of accuracy. In the meantime, statistical checks such as RMSE, MSE, and MAE were performed, as listed in Table 2. The evaluation was undertaken using Equations (1)–(3)):

(1) $$\mathrm{RMSE}=\sqrt{\frac{\sum_{i=1}^{n}\;{\left(ex_{i}-mo_{i}\right)}^{2}}{n}}$$

(2) $$\mathrm{MAE}=\frac{\sum_{i=1}^{n}|ex_{i}-mo_{i}|}{n}$$

(3) $$R=\frac{\sum_{i=1}^{n}\left(ex_{i}-{\bar{ex}}_{i}\right)\left(mo_{i}-{\bar{mo}}_{i}\right)}{\sqrt{\sum_{i=1}^{n}{\left(ex_{i}-{\bar{ex}}_{i}\right)}^{2}\sum_{i=1}^{n}{\left(mo_{i}-{\bar{mo}}_{i}\right)}^{2}}}$$

where $ex_{i}$, $mo_{i}$, ${\bar{ex}}_{i},\;{\bar{mo}}_{i},$ and *n* are the practical, anticipated, mean practical, and mean anticipated values and sample size, respectively.

In order to compare the CV of each selected model with their outcomes, as shown in Table 2, the RMSE, R^2^, and MAE were evaluated. As demonstrated in Table 1, an increased error value in the RF model results in a higher coefficient of determination (R^2^) value, demonstrating that the RF technique is more precise than both the DT and ANN models. Table 3 shows the details of the analysis used in the CV procedure. Statistical checks were also applied to the DT, ANN, and RF approaches, as shown in Table 3. The lesser error value suggests a stronger correlation coefficient (R^2^). Several coefficients, including the RMSE, R^2^, MAE, and MSE, were explored for use in the evaluation of the k-fold CV and the distributions of these correlations for the DT, ANN, and RF models. The RF algorithm with the lowest error value and the highest R^2^ value demonstrated significant precision in outcome prediction. The greatest, minimum, and average R^2^ values for the DT algorithm were 0.97, 0.17, and 0.61, respectively. The RF model’s maximum, minimum, and average R^2^ values were 0.98, 0.26, and 0.69, respectively, while the equivalent values for the ANN model were 0.94, 0.05, and 0.60, respectively.

## 5. SHAP Analysis for the Effect of Raw Materials

Typically, the best description of a basic model, such as linear regression, is the model itself. However, it is challenging to describe how complicated models, such as machine-learning models, function, and how input values affect key parameters. By using the Shapley value, the SHAP, which is focused on game theory and local explanations, can explain the relationship between inputs and targets. The random forest method produced the most accurate prediction model for the STS of the concrete, including recycled aggregate. Consequently, the model interpretation for the STS of the RCA concrete was developed using SHAP analysis.

**Figure 13:**
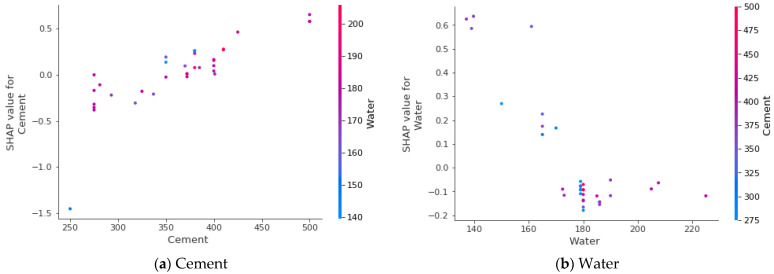

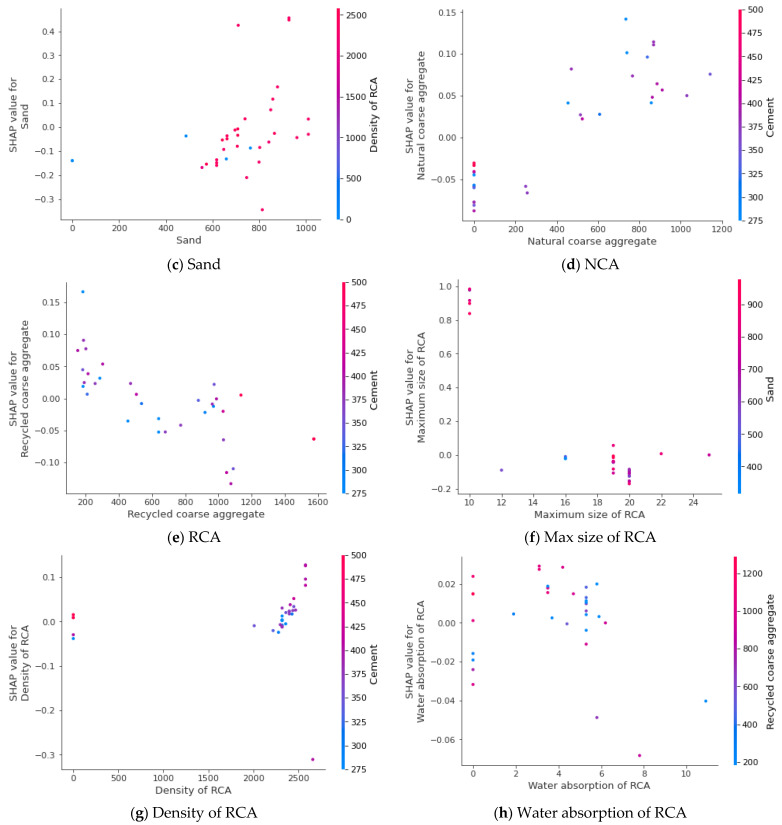
Effect of raw material on STS of recycled aggregate concrete.

Figure 13 depicts the feature interactions for the STS of the RCA concrete. Figure 13a illustrates the cement feature interaction. As shown in Figure 13a, the cement interacted positively with the STS of the concrete. The plot shows that increasing the cement content resulted in a higher STS of the RCA concrete. Figure 13b depicts the negative interaction of the water with the STS of the concrete. It is a clear fact that an increase in water results in a decrease in concrete’s strength. Similarly, Figure 13c shows the interaction of the sand with the STS of the selected concrete. It was noted from the distribution that the increase in the amount of sand showed a positive direct relation to the concrete’s strength in the range of 600 kg/m^3^ to 100 kg/m^3^. Figure 13d indicates the interaction of the natural coarse aggregate with the STS of the concrete, showing the linear trend toward and positive direct relation with the strength of the concrete. However, Figure 13e,f shows the interaction of the recycled coarse aggregate and the maximum size of the recycled coarse aggregate with the strength of concrete. Both of the plots demonstrate the negative impact on the output. This was because of the high porosity of the recycled aggregate, which affected the strength of concrete. Moreover, as the density of the RCA increased, it showed a direct positive relation to the STS of the concrete, as can be seen in Figure 13g. This was due to the fact that, as the density increased, the material became less porous, which reduced the absorption of the water, leading to higher strength. However, Figure 13h also shows both the positive and the negative interactions. On the plot, water absorption of up to 4% indicates the increment towards the STS of the concrete, while beyond 4%, a decrease in the trend towards the STS of the concrete can be seen. This was because if the recycled aggregate absorbed more water from the mix, the amount of water required for cement hydration decreased, which affected the strength of the concrete material.

## 6. Sensitivity Analysis

**Figure 14:**
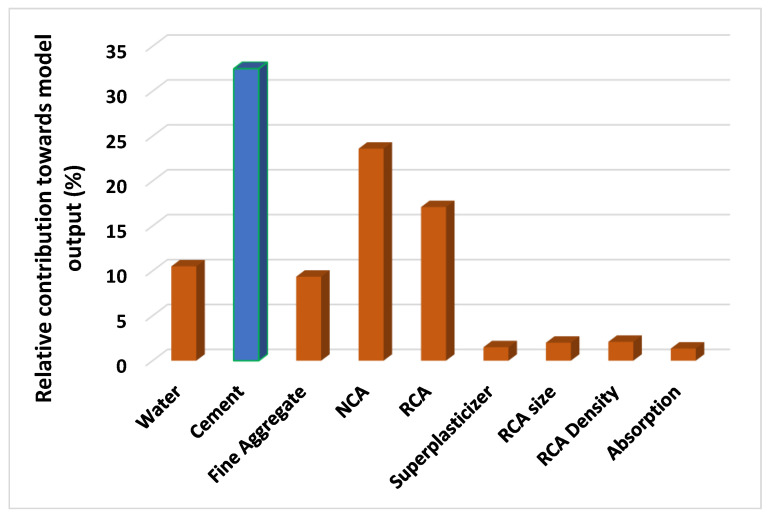
Input variables’ contribution level in percentages to the anticipation of the outcome.

In this strategy, Figure 14 shows how different factors affect the prediction of the STS of RA concrete. According to the report, the cement was the highest contributor to the predictions, with 32.5 percent, followed by NCA, with 23.6 percent, and RA, with 17.1 percent. The prediction of the STS of the RA-based concrete was least affected by the fine aggregate, water, superplasticizers, coarse aggregate size, RCA density, and RCA water absorption.

## 7. Discussion

This study demonstrates the utility of AI approaches for estimating the STS of recycled aggregate concrete (RCA). Due to the low tensile strength and brittle character of concrete material, it is typically not assumed that concrete can sustain direct tension. The STS of concrete is one of the most fundamental and significant qualities that significantly influence the extent and size of structural cracks. The split-cylinder test is an indirect method of determining the tensile strength of concrete. The STS was selected for investigation in this study because of its importance and the relative lack of research on it, as opposed to the compressive and flexural strength of concrete.

**Figure 15:**
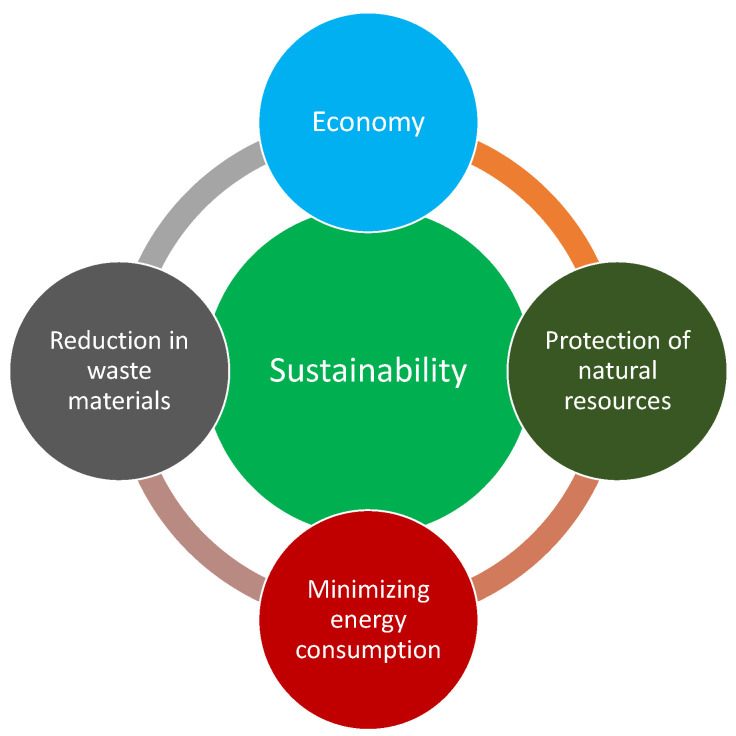
Schematic representation of the numerous sustainability-related enhancements.

The incorporation of RA into concrete is crucial for developing sustainable concrete. This strategy contributes not only to the minimization of waste on the planet but also to a robust economy, the protection of natural resources, and the decrease in energy consumption. Figure 15 depicts a graphical depiction of several sustainability-related criteria.

**Figure 16:**
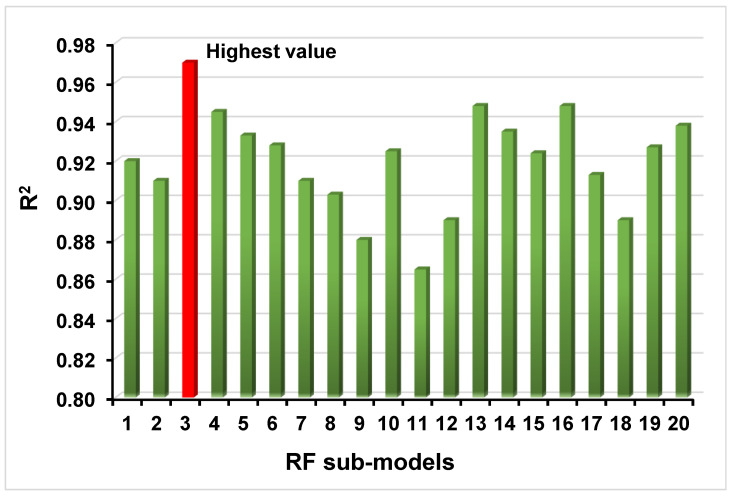
Results of RF sub-models indicating the values for every model’s significance level.

RF is utilized in supervised learning to eliminate both bias and variance. The theory is based on the notion that individuals develop sequentially. Except for the initial learner, all succeeding learners are developed from prior learners. In a sense, learners become powerful. For its part, replacement sampling, RF is an approach used to randomly select data from a set, with the added feature that the same data point might be taken more than once. Using a variety of data samples, these weak models were trained separately and based on the task at hand (regression or classification, for example); the average or majority of the projections result in a more accurate assessment. This was performed in order to establish which algorithm performed better in terms of forecasting performance than all others. The RF model’s output was more valuable, with an R^2^ value of 0.97 compared to 0.85 for the DT model and 0.86 for the ANN model, indicating a higher level of precision. In comparison, Yuan et al. [58] also predicted the compressive and flexural strength of recycled aggregate concrete. Their results were also within the acceptable range and confirmed that these techniques can be successfully employed to investigate the mechanical properties of concrete. In addition, we analyzed the performance of the DT, ANN, and RF models by a statistical approach and the CV method, which we found to be effective. When the error levels are low, the model’s performance is satisfactory. However, evaluating and recommending the optimal machine-learning regression model for calculating probability over a broad range of topics is challenging due to the fact that the model’s performance is highly dependent on its input parameters and data points. Fortunately, there is a solution. Ensemble machine-learning methods, on the other hand, frequently take advantage of weak learners by creating sub-models that can be trained on data and optimized for the maximum R^2^ value. Figure 16 depicts the distribution of the R^2^ values for RF sub-models based on their frequency. Furthermore, according to the research, RF models surpass other machine-learning techniques in terms of accuracy and precision. In addition, we performed a sensitivity analysis to examine the impact of each input parameter on the expected STS (see Additional Resources). The input parameter values, and the number of data points used in the model can both have an impact on the model’s performance and accuracy.

## 8. Conclusions

This study aimed to illustrate how the soft-computing algorithms from machine learning can be utilized to predict the strength (STS) of recycled aggregate concrete. The DT, ANN, and RF methods were applied to anticipate the STS of the RA-based concrete. The following inferences were drawn:

- The RF model outpaced both the DT and the ANN techniques in terms of prediction accuracy, as demonstrated by a higher coefficient of determination (R^2^) and lower error values. The DT, ANN, and RF models were found to have R^2^ values of 0.85, 0.86, and 0.97, respectively.
- In addition, the statistical analysis and the k-fold cross-validation method demonstrated that all of the applied approaches (GEP, ANN, and RF) functioned adequately. In addition, these tests indicated that the RF model performed better than the DT and ANN models.
- The analysis of the sensitivity showed that the most influential component (cement) effectively (32.50 percent) anticipated the required output, as opposed to the other variables used as input parameters.
- AI approaches can be successfully utilized to investigate the properties of materials based on the given variables in a limited time period.
- AI approaches enable more accurate predictions of material properties’ attributes without requiring sample production or experimental testing [59].

Moreover, it is further suggested that the data points can be increased to check the performance of selected algorithms. In addition, the input parameters, such as temperature, humidity, and size of the material, can also be increased to investigate the precision levels of the models.

## Figures and Tables

**Table 1:** Expressive inspection of the input variables.

Parameters	WA	CE	FA	NACA	RECA	SPS	SRA	DRA	WARA
Mean values	180.38	364.42	688.47	382.02	656.69	1.11	18.29	2081.07	4.56
Standard error	1.41	5.49	17.68	30.72	29.34	0.15	0.29	62.64	0.22
Median value	180.00	372.00	715.00	395.50	577.50	0.00	20.00	2360.00	5.30
Mode value	180.00	380.00	0.00	0.00	1135.40	0.00	20.00	2320.00	5.30
Standard deviation	18.17	70.73	227.85	395.77	377.99	1.88	3.80	807.11	2.87
Sample variance	330.11	5003.37	51,917.40	156,630.68	142,876.62	3.55	14.41	651,425.17	8.23
Range	88.00	442.00	1010.00	1168.00	1517.30	7.80	15.00	2661.00	10.90
Lower values	137.00	158.00	0.00	0.00	57.00	0.00	10.00	0.00	0.00
High values	225.00	600.00	1010.00	1168.00	1574.30	7.80	25.00	2661.00	10.90
Total sum	29,942.99	60,493.00	114,285.63	63,414.57	109,011.35	183.49	3036.00	345,457.00	757.10
Count	166.00	166.00	166.00	166.00	166.00	166.00	166.00	166.00	166.00

**Table 2:** Results of the CV of k-fold for the employed models.

DT				RF			ANN		
K-Fold	MAE	RMSE	R^2^	MAE	RMSE	R^2^	MAE	RMSE	R^2^
1	0.91	0.95	0.91	0.49	0.67	0.37	0.92	1.15	0.08
2	0.80	0.88	0.75	0.71	0.88	0.65	0.83	1.09	0.62
3	0.51	0.65	0.26	0.63	0.59	0.96	0.48	0.73	0.77
4	0.79	1.20	0.39	0.74	1.04	0.98	0.91	1.19	0.93
5	0.19	0.15	0.76	0.63	0.34	0.86	0.14	0.17	0.94
6	0.42	0.49	0.95	1.02	0.38	0.74	0.40	0.55	0.05
7	1.10	1.14	0.97	0.83	0.73	0.72	1.19	1.20	0.24
8	0.69	1.07	0.37	0.63	0.81	0.26	0.78	1.11	0.57
9	0.83	0.79	0.17	0.52	0.66	0.55	0.87	0.87	0.88
10	0.18	0.47	0.55	0.98	0.99	0.77	0.28	0.50	0.90

**Table 3:** Results of the employed checks.

ML Approaches	RMSE (MPa)	MAE (MPa)	MSE (MPa)
DT	0.365	0.308	0.133
RF	0.166	0.143	0.028
ANN	0.375	0.315	0.141

## Data Availability

The data used for the development of the models are reported in the paper.
